# The effect of prescribed burning on plant rarity in a temperate forest

**DOI:** 10.1002/ece3.3771

**Published:** 2018-01-08

**Authors:** John Patykowski, Greg J. Holland, Matt Dell, Tricia Wevill, Kate Callister, Andrew F. Bennett, Maria Gibson

**Affiliations:** ^1^ School of Life and Environmental Sciences Deakin University Geelong Vic. Australia; ^2^ Department of Ecology, Environment and Evolution La Trobe University Bundoora Vic. Australia; ^3^ Ecology Australia Pty Ltd Fairfield Vic. Australia; ^4^ Arthur Rylah Institute for Environmental Research DELWP Heidelberg Vic. Australia

**Keywords:** Australia, burn season, controlled and prescribed burning, experiment, landscape disturbance, rainfall, wildfire

## Abstract

Rare species can play important functional roles, but human‐induced changes to disturbance regimes, such as fire, can inadvertently affect these species. We examined the influence of prescribed burns on the recruitment and diversity of plant species within a temperate forest in southeastern Australia, with a focus on species that were rare prior to burning. Floristic composition was compared among plots in landscapes before and after treatment with prescribed burns differing in the extent of area burnt and season of burn (before–after, control‐impact design). Floristic surveys were conducted before burns, at the end of a decade of drought, and 3 years postburn. We quantified the effect of prescribed burns on species grouped by their frequency within the landscape before burning (common, less common, and rare) and their life‐form attributes (woody perennials, perennial herbs or geophytes, and annual herbs). Burn treatment influenced the response of rare species. In spring‐burn plots, the recruitment of rare annual herbs was promoted, differentiating this treatment from both autumn‐burn and unburnt plots. In autumn‐burn plots, richness of rare species increased across all life‐form groups, although composition remained statistically similar to control plots. Richness of rare woody perennials increased in control plots. For all other life‐form and frequency groups, the floristic composition of landscapes changed between survey years, but there was no effect of burn treatment, suggesting a likely effect of rainfall on species recruitment. A prescribed burn can increase the occurrence of rare species in a landscape, but burn characteristics can affect the promotion of different life‐form groups and thus affect functional diversity. Drought‐breaking rain likely had an overarching effect on floristic composition during our study, highlighting that weather can play a greater role in influencing recruitment and diversity in plant communities than a prescribed burn.

## 1 INTRODUCTION

Managing disturbance regimes in ecosystems is crucial for avoiding the loss of rare and endemic species and homogenizing species composition (McKinney & Lockwood, 1999). Although ecosystem processes often are maintained primarily by a low proportion of the total species—the dominants—in any given community (Sasaki & Lauenroth, 2011; Schwartz et al., 2000; Smith & Knapp, 2003), it is increasingly evident that rare species can play unique (Jain et al., 2014; Leitão et al., 2016; Mouillot et al., 2013) or keystone roles (Marsh, Arnone, Bormann, & Gordon, 2000) or collectively make important contributions to ecosystem function over different temporal or spatial scales (Allan et al., 2011; Lyons, Brigham, Traut, & Schwartz, 2005; Lyons & Schwartz, 2001). Consequently, the loss of rare species—those with low abundance or limited distribution (Gaston, 1994; Rabinowitz, 1981)—can result in a reduction in the functional trait diversity of communities (Leitão et al., 2016). The loss of physiological, morphological, and phenological attributes that determine how species respond to environmental factors (Pérez‐Harguindeguy et al., 2013) can render ecosystems more vulnerable to collapse (MacDougall, McCann, Gellner, & Turkington, 2013).

Climate change is predicted to bring larger fluctuations in weather patterns, including more severe drought (Smith et al., 2009), and an associated increase in the intensity, duration, and extent of wildfires in many parts of the world (Batllori, Parisien, Krawchuk, & Moritz, 2013; Moritz et al., 2012). In fire‐prone ecosystems, prescribed burning often is used as a management tool to reduce fuel loads and protect human life and assets from wildfire (Penman et al., 2011). As a large‐scale disturbance process, the effects of prescribed burning need to be understood at the landscape scale, but the difficulty of carrying out manipulative experiments at this scale means that such knowledge is limited (Driscoll et al., 2010), especially for rare species.

Multiple aspects of a fire regime can influence the status of plant species. The burn season (timing) affects the response of species and the trajectory of community recovery because of interspecific differences in phenology, life‐history, and physiology (Drewa, Platt, & Moser, 2002; Gill, 1975). The extent, intensity, and duration of a fire also strongly affect how plants respond, with these properties being heavily influenced by the season of burn, weather preceding the burn, and fuel‐load characteristics (Bradstock, Williams, & Gill, 2012). In temperate and Mediterranean‐type climates, ambient temperatures are lower and the vegetation wetter in autumn, winter, and spring, resulting in less intense fires that typically burn a lower proportion of the vegetation than fires occurring in summer (Sullivan, McCaw, Cruz, Matthews, & Ellis, 2012). Consequently, prescribed burns generally are conducted in autumn and spring when fires are easier to control. Burning in autumn may produce less intense burns than spring, reducing pressure on species that resprout after disturbance. However, soil temperatures in an autumn burn may not reach levels needed to break morphological or physiological dormancy in soil‐stored seed, and thus the response of rare species present only in the soil seed bank may be lower in autumn than spring.

In this study, we used a landscape‐scale, manipulative experiment in a box–ironbark eucalypt forest in southeastern Australia to examine the short‐term effects of prescribed burning on the plant community. Our specific focus was on the response of species that were rare prior to vegetation being burnt (recorded in <5% of study plots), and the life‐form groups (woody perennials, perennial herbs or geophytes, and annual herbs) to which these species belonged. As the fire responses of a species must be in context with the responses of other species within a system, the relative influence of a prescribed burn on all plant species was examined. Box–ironbark forests are not prone to regular widespread fire (Calder & Calder, 2002), nor are they dependant on fire for their regeneration (Cheal, 2010). However, many plants found in box–ironbark forests are fire‐adapted, either successfully resprouting after fire (Morgan, 1999), having fire‐cued seed germination (Tolsma, Cheal, & Brown, 2007), or both. Some species may require infrequent fire for sufficient recruitment events to maintain viable populations, without which a species may appear rare or absent (Duff, Bell, & York, 2013; Nield, Ladd, & Yates, 2009), although a viable soil seed bank may remain (Davies, Whalen, Mackay, Taylor, & Pisanu, 2013;).

Severe rainfall deficit affected southeastern Australia in a period known as the Millennium Drought between 1997 and 2009 (Gergis et al., 2012). This drought negatively affected recruitment (Meers & Adams, 2003) and vegetation structure (Bennett et al., 2013) in box–ironbark forests. The drought broke in early 2010, at the time of initial floristic surveys. Owing to the inclusion of control landscapes, we were able to compare the effect of rainfall versus the effect of prescribed fire and rainfall on plant recruitment. Specifically, we asked:

- Does prescribed burning increase floristic diversity in excess of rainfall‐driven, background recruitment?
- Does prescribed burning influence the floristic composition of sites? Are there different responses from species assigned to different life‐form groups (woody perennials, perennial herbs or geophytes, and annual herbs) or frequency groups (rare, less common, and common)?
- Are the contributions of rare, less‐common, and common species to the floristic composition of the vegetation influenced by prescribed burning?

## 2 MATERIALS AND METHODS

### 2.1 Study area

**Figure 1:**
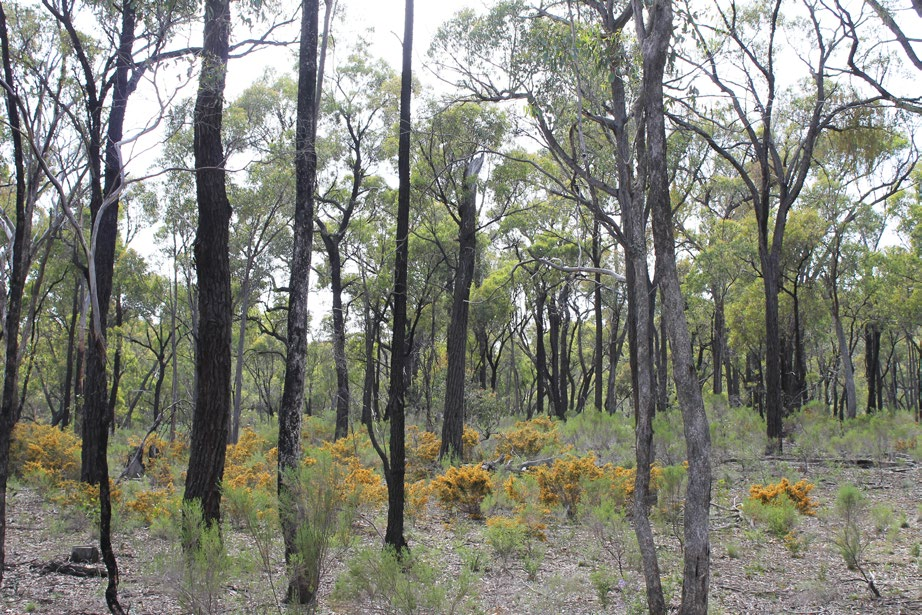
Typical box–ironbark vegetation within the Heathcote‐Graytown‐Rushworth forest, with an open canopy dominated by *Eucalyptus tricarpa* (red ironbark)*, Eucalyptus microcarpa* (gray box) and a sparse, shrubby understory (J. Patykowski)

Surveys were conducted in the Heathcote‐Graytown‐Rushworth forest, the largest remaining box–ironbark forest in Victoria, Australia, covering an area of approximately 40,000 ha (Environment Conservation Council; ECC 2001). These forests were extensively cleared for deep lead alluvial mining and agriculture in the mid‐1800s and, while regenerating over the last century, have been subject to ongoing selective logging (Muir, Edwards, & Dickins, 1995; Newman, 1961). The dry, sclerophyll forest is characterized by an open eucalypt overstorey up to 20 m tall, with a sparse to well‐developed understory of small trees and shrubs, and a range of herbs and grasses (Figure 1). The dominant canopy species are *Eucalyptus tricarpa* (L.A.S.Johnson) L.A.S.Johnson & K.D.Hill (red ironbark) and *Eucalyptus microcarpa* (Maiden) Maiden (gray box), with *Eucalyptus polyanthemos* Schauer (red box) and *Eucalyptus macrorhyncha* F.Muell. ex Benth. (red stringybark) occurring on drier slopes. Soils are typically stony and shallow or skeletal clay loams of low fertility, derived from Lower Paleozoic sandstone and slates, interbedded with quartz reefs (Douglas & Ferguson, 1988; Mikhail, 1976). The region is characterized by peneplains and low, gently undulating hills, ranging in elevation between 150 and 300 m (Douglas & Ferguson, 1988).

The climate is temperate; mean daily maximum temperatures are greatest in January (~29°C) and coolest in July (~12°C) (Bureau of Meteorology 2015; Redesdale weather station, station ID: 088051). Mean annual rainfall is ~594 mm; August is the wettest month (~69 mm) and February the driest (~31 mm) (Bureau of Meteorology 2015; Heathcote weather station, station ID: 088029). During the Millennium Drought (1997–2009), mean annual rainfall was 480 mm, compared with 869 mm for 2010–2011 when the drought broke (Bureau of Meteorology 2015).

### 2.2 Experimental prescribed burns

**Figure 2:**
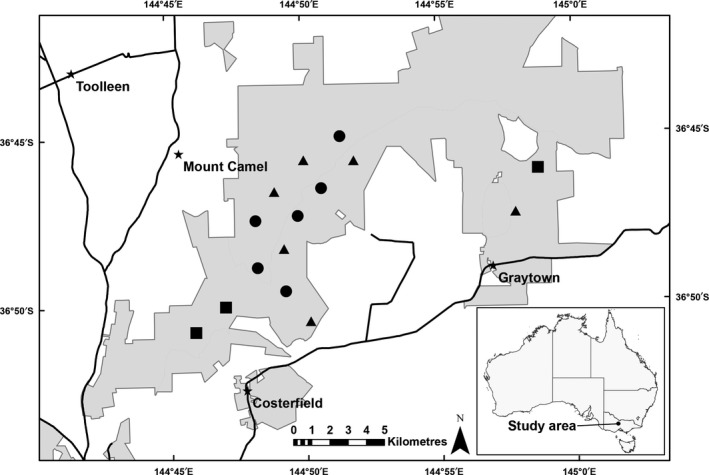
Location of study landscapes in the Heathcote‐Graytown‐Rushworth forest, in southeastern Australia. ■ unburnt reference (control), ● autumn burn, and ▲ spring burn

Data were collected from 15 study landscapes (Figure 2) as part of a broader study examining the effects of experimental prescribed burns on a range of ecological attributes at a landscape scale (Holland, Clarke, & Bennett, 2017). Study landscapes were ~70–120 ha in area, separated by >500 m, and bounded by management tracks to enable prescribed burns to be contained. None of the study landscapes had experienced burning for at least 30 years, and areas recently subjected to timber harvesting (approximately <20 years) were avoided.

The study design involved three treatment groups: landscapes to be burned in autumn (*n* = 6), landscapes to be burned in spring (*n* = 6), and unburned reference landscapes to serve as experimental controls (*n* = 3). Burn treatment was randomly assigned to the landscape. Prescribed burns were conducted by fire management agencies (Department of Environment, Land, Water and Planning, and Parks Victoria) in autumn (late February–April) and spring (October–November) of 2011. The mean burn cover achieved for autumn‐burn landscapes was 26% (range 22%–51%) and for spring‐burn landscapes 69% (range 52%–89%) (Holland et al., 2017).

### 2.3 Pre‐ and postburn floristic surveys

Eight permanent 20 m × 20 m plots were established in each study landscape to monitor vegetation composition (total *n* = 120). In each plot, five 1 m × 1 m quadrats were established at fixed locations and surveyed for plant species presence/absence (total *n* = 600). These data were used to determine the frequency of occurrence for all native vascular plant species per 20 m × 20 m plot. Species that occurred within, or had foliage overhanging all five quadrats, were given a frequency score of 1; species occurring within, or had foliage overhanging four, three, or two quadrats, received a score of 0.8, 0.6, or 0.4, respectively. Species occurring within, or had foliage overhanging a single quadrat, or the 20 m × 20 m plot (but not in a quadrat), were given a frequency score of 0.2.

Preburn surveys were conducted in each of the study landscapes during spring (September–October) of 2010. This was just after the Millennium Drought broke; rainfall from January to September of 2010 was 597 mm, with 979 mm falling by the end of 2010 (nearly double the long‐term average). Annual rainfall was above average in 2011 (759 mm), and below average in 2012 and 2013 (518 and 521 mm, respectively; Bureau of Meteorology 2015). All study landscapes were resurveyed in spring 2013, which allowed time for species to germinate in the case of a delayed germination response to fire (Ooi, Auld, & Whelan, 2004) and to become established. This both aided species identification and affirmed their contribution to ecosystem function (i.e., germinants that failed to successfully recruit would make little, if any, functional contribution).

Plants that could not be identified to species level, or recognized as a distinct species, were not included for analysis. Species in the family Poaceae, Orchidaceae, and genus *Arthropodium* R. Br. were excluded as many species within each family and genus have similar leaf characteristics, and often lacked reproductive material necessary for identification to a species level. Species were categorized into life‐form groups: woody perennials, perennial herbs or geophytes, or annual species.

### 2.4 Categorizing species frequency of occurrence

Species were categorized into groups based on their presence among 20 m × 20 m study plots in 2010, prior to burning. Species present in ≤5% of plots (six or fewer plots) were classified as “rare,” regardless of their frequency score, as they had a limited distribution within the study area. Species were considered “common” if present in ≥50% of the plots, and their mean frequency score was ≥0.25 within the plots they occupied: This ensured species classified as common were both widespread and relatively abundant. Species present in 5–50% of the plots were classified as “less common.”

### 2.5 Data analysis

First, to examine whether there was an effect of survey year and burn treatment on overall floristic diversity at the landscape scale, we calculated the effective number of species for each study plot (the exponential of Shannon H' diversity index; Hill, 1973), using PRIMER ver. 7 (PRIMER‐E Ltd, Plymouth, UK). Differences among treatments were compared using permutational analysis of variance (PERMANOVA) of Euclidean distances between study plots (two‐way design with year and burn treatment as fixed effects). We used the PERMANOVA+ package in PRIMER for these tests and set an a priori significance level of α = 0.05.

Second, we used a two‐way PERMANOVA to test the effect of survey year and burn treatment (fixed effects), and their interaction, on floristic composition. We included “landscape” as a random factor in the analysis, nested within burn treatment, to account for repeated sampling of landscapes through time. A significant interaction effect would indicate that changes between survey years differed between the burn treatments. These tests were undertaken for the floristic community as a whole, and separately for each plant life‐form (i.e., woody perennials, perennial herbs or geophytes, and annual herbs) and frequency group (i.e., rare, less common, and common). Similarity matrices of floristic composition among plots were first created by using the Bray–Curtis index on untransformed frequency data. When creating similarity matrices for annual herbs and rare species, a “dummy species” was added as present in each plot to cope with a prevalence of absences (Clarke, Somerfield, & Chapman, 2006). Type III (partial) sum of squares was used, and floristic data were permuted 9,999 times under a reduced model. Permuting floristic composition within treatments assumes that the observation units are exchangeable under a true null hypothesis and breaks down any temporal or spatial correlation, if present (Anderson, 2001).

Where there was a significant interaction between survey year and burn treatment, pair‐wise comparisons were made between each burn treatment and year combination using PERMANOVA, thus identifying where differences between treatment groups occurred. We also compared species richness for each life‐form group between survey years, within each burn treatment, using Wilcoxon signed‐rank tests for paired, nonparametric data.

An assumption of PERMANOVA is that dispersion of samples *within* treatment groups is homogenous *among* treatment groups (Anderson, 2006); we tested this using the PERMDISP routine in the PERMANOVA+ package in PRIMER (Anderson, Gorley, & Clarke, 2008). Where differences in dispersion were detected, unconstrained ordination plots (nonmetric multidimensional scaling; nMDS) were generated to determine if differences detected by PERMANOVA were likely caused by differences in grouping among treatments, as an effect of dispersion among treatments, or a combination of both (Anderson et al., 2008).

Third, we determined the similarity in floristic composition of study landscapes *within* survey years, and the dissimilarity of floristic composition *between* survey years, by using the one‐way similarity percentages (SIMPER) routine with the Bray–Curtis similarity index, performed in PRIMER. The collective percentage contribution of rare, less‐common, and common species in each life‐form group was calculated within and between survey years.

Finally, we used the SIMPER routine, with rare species only, to determine the contribution of each life‐form group to the similarity in floristic composition of rare species in landscapes *within* each burn treatment, for each survey year. We then calculated the contribution of each life‐form group to the dissimilarity in floristic composition of rare species *between* burn treatments for each survey year.

Nonmetric multidimensional scaling based on Bray–Curtis similarity between sites was used to construct two‐dimensional ordination diagrams displaying the location of study plots within each treatment group relative to one another, with 500 restarts per plot.

## 3 RESULTS

One hundred and twenty‐one vascular plant species were identified during the study (not including species in the families Poaceae, Orchidaceae, or genus *Arthropodium*), with a total of 98 species in 2010 and 118 species in 2013. There were 11 common, 48 less‐common, and 39 rare species identified in the preburn 2010 survey. Three species recorded in 2010 were not recorded in 2013, and 23 species were recorded in 2013 that were not recorded in 2010.

### 3.1 Effects of prescribed burns on floristic diversity

**Figure 3:**
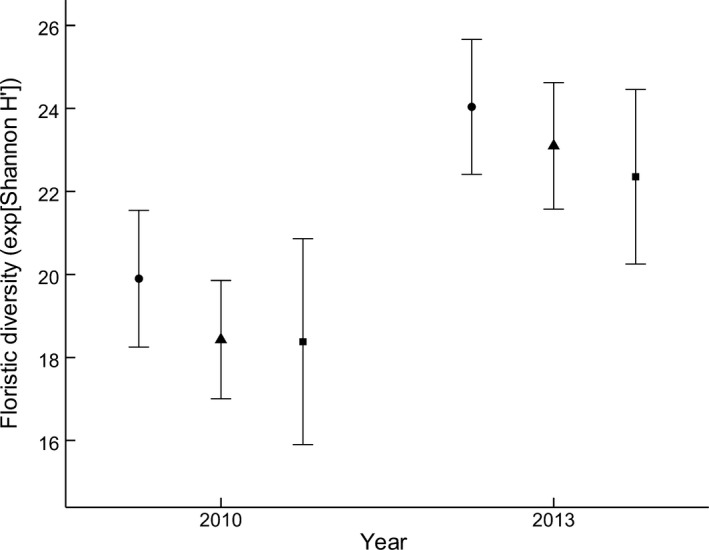
Mean (±1.96 *SE*) floristic diversity (effective number of species; exponential of Shannon H′ diversity index) of study landscapes before (2010) and after (2013) experimental burns conducted during different seasons in a box–ironbark forest in southeastern Australia; ■ unburnt reference (control), ● autumn burn, and ▲ spring burn

Floristic diversity (effective number of species) differed between 2010 and 2013 (pseudo‐*F*_1,2_ = 31.75, *p *<* *.001); preburn landscapes (2010) had a lower floristic diversity than postburn landscapes (2013; Figure 3). Floristic diversity did not differ among burn treatments (pseudo‐*F*_1,2_ = 1.74, *p *=* *.17), and there was no interaction between burn treatment and survey year (pseudo‐*F*_1,2_ = 0.08, *p *=* *.91). Dispersion among treatment groups was homogenous (*F*_5,234_ = 0.45, *p *=* *.82).

### 3.2 Effects of prescribed burns on floristic composition

**Figure 4:**
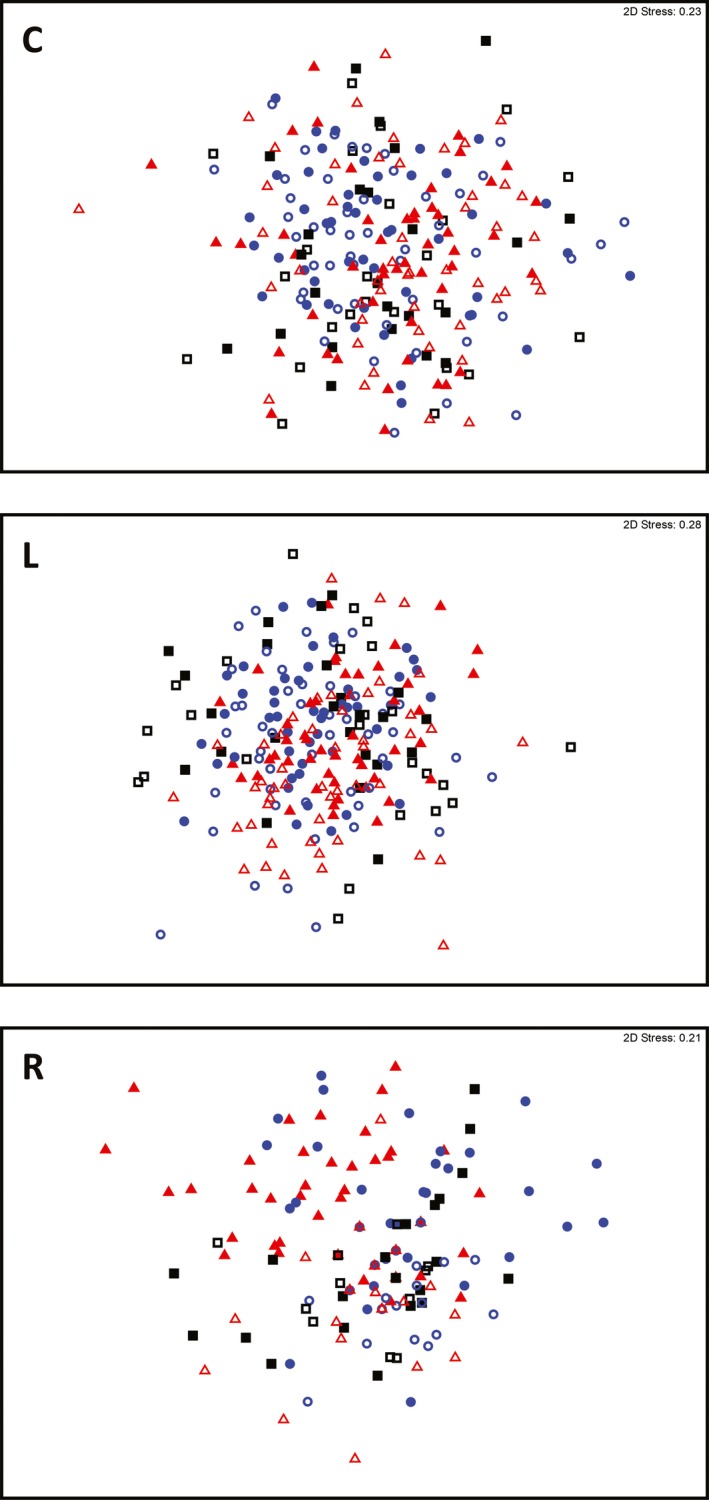
Ordination (nMDS) of plots within a box–ironbark forest in southeast Australia, based on the floristic composition of common (C), less common (L), and rare (R) species before (2010; open symbols) and after (2013, closed symbols) experimental prescribed burns conducted during different seasons; ■ unburnt reference (control), ● autumn burn, and ▲ spring burn

**Figure 5:**
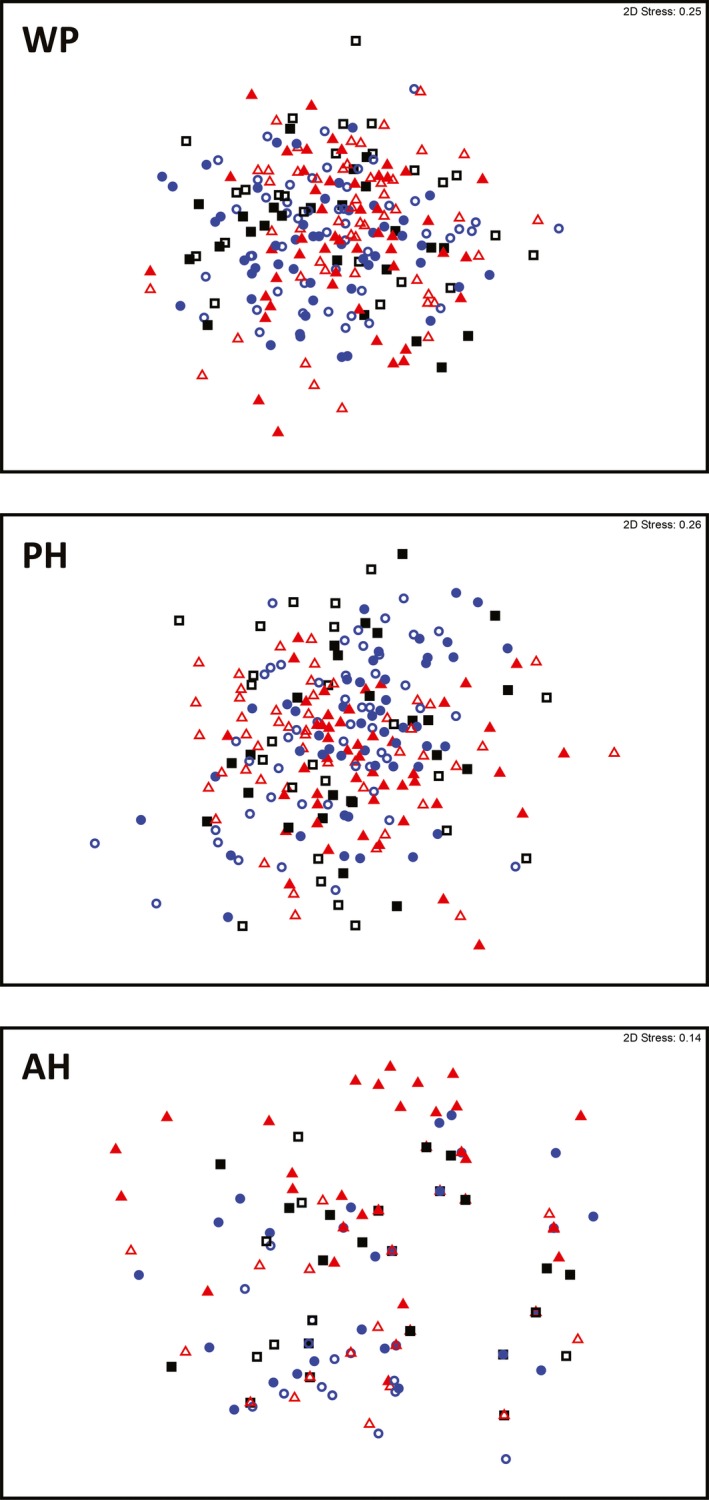
Ordination (nMDS) of plots within a box–ironbark forest in southeast Australia, based on the floristic composition of woody perennial (WP), perennial herb or geophyte (PH), and annual herb (AH) species before (2010; open symbols) and after (2013, closed symbols) experimental prescribed burns conducted during different seasons; ■ unburnt reference (control), ● autumn burn, and ▲ spring burn

PERMANOVA analysis indicated that overall floristic composition differed between 2010 and 2013, as did the composition of plant life‐form and frequency of occurrence groups (Table 1). The effect of year appeared to be strongest for rare species and annual herbs; for all other groups, there was a high degree of overlap in floristic composition between survey years (Figures 4 and 5). Burn treatment generally did not affect floristic composition (Table 1). There was a random effect of landscape; study plots within some landscapes displayed a degree of aggregation within the multidimensional space, based on their floristic composition (Figures S1–S7). A significant interaction between survey year and burn treatment occurred only for the composition of rare species (pseudo‐*F*_1,2_ = 2.0, *p *=* *.004). Thus, rare species was the only group to show a clear effect of burn treatment on floristic composition, after taking into account differences between survey years. The floristic composition of rare species in 2010 (preburning) did not differ among landscapes. However, in 2013, after the prescribed burns, the composition of rare plants in the spring‐burn landscapes (more extensive burn cover) differed from that of autumn‐burn landscapes, and separation of spring‐burn from unburnt control landscapes occurred (Table 2; Figure 4).

**Table 1:** PERMANOVA results for the effect of survey year (2010, 2013) and burn treatment (unburnt control, autumn, spring) on the floristic composition of landscapes (LS, included as a random factor, nested within burn treatment). Comparisons are provided with respect to plant species' life‐form and frequency groups. Significant values are provided in bold

Group	Year		Burn		LS (Burn)		Year*Burn		Year*LS(Burn)	
	Pseudo‐*F*	*p*	Pseudo‐*F*	*p*	Pseudo‐*F*	*p*	Pseudo‐*F*	*p*	Pseudo‐*F*	*p*
All species	12.01	**<.001**	0.71	.854	5.77	**<.001**	1.02	.443	0.79	.981
Life‐form										
Woody perennials	16.19	**<.001**	0.56	.929	6.08	**<.001**	1.23	.294	0.57	.999
Perennial herbs or geophytes	6.23	**<.001**	0.93	.526	5.36	**<.001**	0.87	.573	1.06	.339
Annual herbs	12.10	**<.001**	0.57	.738	3.95	**<.001**	1.00	.427	1.19	.188
Frequency										
Common	6.75	**.002**	0.54	.870	4.93	**<.001**	0.60	.701	0.48	.999
Less common	11.91	**<.001**	0.72	.845	6.18	**<.001**	1.13	.344	0.89	.805
Rare	13.46	**<.001**	1.22	.221	3.72	**<.001**	1.97	**.034**	1.19	.062

**Table 2:** Pair‐wise PERMANOVA comparisons of the similarity in floristic composition of rare plant species among landscapes assigned to different burn treatments, in 2010 (before prescribed burns), and in 2013 (after burns). Significant values are provided in bold

Pair‐wise comparison	*t*	*p*
2010 surveys		
Control, Autumn	1.08	.239
Control, Spring	0.89	.545
Autumn, Spring	1.14	.223
2013 surveys		
Control, Autumn	0.82	.845
Control, Spring	1.41	.095
Autumn, Spring	1.37	**.049**

Dispersion among treatment groups was homogenous between 2010 and 2013 for overall floristic composition, woody perennials, perennial herbs, and common species groups (Table S1). Dispersion among plots also was homogenous among all burn treatments, except when including only less‐common species (Table S1).

In the annual herbs group, dispersion of plots differed between 2010 and 2013 (*F*_1,238_ = 10.52, *p *=* *.005), and between plots pre‐ and postburn in the autumn treatment group (*t *=* *3.29, *p *=* *.003; Table S2). The nMDS ordination (Figure 5) indicated separation of autumn and spring‐burn treatment groups between years, and an effect of dispersion (the average distance between autumn plots increased in 2013; Table S3); that is, floristic composition changed between survey years, *and* there was an effect of dispersion (autumn‐burn plots became less similar in 2013).

In the less‐common frequency group, dispersion differed between years (*F*_1,238_ = 13.74, *p *<* *.001), between burn treatments (*F*_1,238_ = 5.01, *p *=* *.010), and between control plots and autumn/spring plots in 2010 (*t *=* *3.37, *p *=* *.003 and *t *=* *2.79, *p *=* *.01, respectively; Table S2). Dispersion of control plots also differed between survey years (*t *=* *3.29, *p *=* *.003), as did spring‐burned plots (*t *=* *3.29, *p *=* *.003). The nMDS ordination (Figure 4) displayed no clear change in the clustering of treatment groups, suggesting the composition of less‐common species did not change markedly between survey years, but plots became more similar in 2013 (Table S3).

For rare species, dispersion differed among plots between survey years, but did not differ among treatments within survey years (Table S2). Clustering of burn treatment groups in the nMDS ordination (Figure 4), and average distance between plots in each burn treatment group (Table S3), indicated that burn treatment groups became more dissimilar in 2013 and also changed in composition.

### 3.3 Contribution of species life‐form and frequency groups to floristic composition

Based on SIMPER analysis, common species generally contributed most to the floristic similarity of the vegetation among study landscapes in each survey year when combined across burn treatments; the contribution of rare species was relatively minor compared with that of common and less‐common species (Table 3). However, for each life‐form group, the contribution made by common species to similarity among landscapes was lower in 2013 (postburn) than 2010 (preburn), with a corresponding increase in the contribution of less‐common and rare species. This trend was most pronounced for the “annual herbs” life‐form group (Table 3). In relation to the dissimilarity among landscapes between years, rare species of annual herbs made a substantial contribution to differentiating landscapes between the 2010 and 2013 surveys; 22% of the dissimilarity in annual herbs was explained by rare species, while 33.6% was explained by common species.

**Table 3:** Contribution (%) of species frequency groups (SIMPER analysis), within each life‐form group, to the floristic similarity of vegetation within (sim%), and dissimilarity among (dis%), landscapes surveyed in 2010 and 2013 (C, common; L, less common; and R, rare), combined across burn treatments. Contributions of the three rarity groups to overall (dis)similarity sum to 100%

Survey year	All species				Woody perennials			
	Relative contribution (%)				Relative contribution (%)			
	sim%	C	L	R	sim%	C	L	R
2010	47.1	62.3	37.6	0.1	46.2	42.7	57.2	0.1
2013	47.7	52.1	46.8	1.1	46.2	32.9	66.4	0.7
	**dis%**				**dis%**			
2010, 2013	54.3	33.4	63.6	3.0	55.5	25.2	68.8	6.0

	Perennial herbs or geophytes				Annual herbs			
	Relative contribution (%)				Relative contribution (%)			
	sim%	C	L	R	sim%	C	L	R
2010	50.5	80.3	19.6	0.1	23.0	84.8	14.7	0.5
2013	53.2	73.8	25.5	0.7	18.8	22.4	67.1	10.5
	**dis%**				**dis%**			
2010, 2013	49.4	45.3	47.6	7.1	84.9	33.6	42.1	22.3

**Figure 6:**
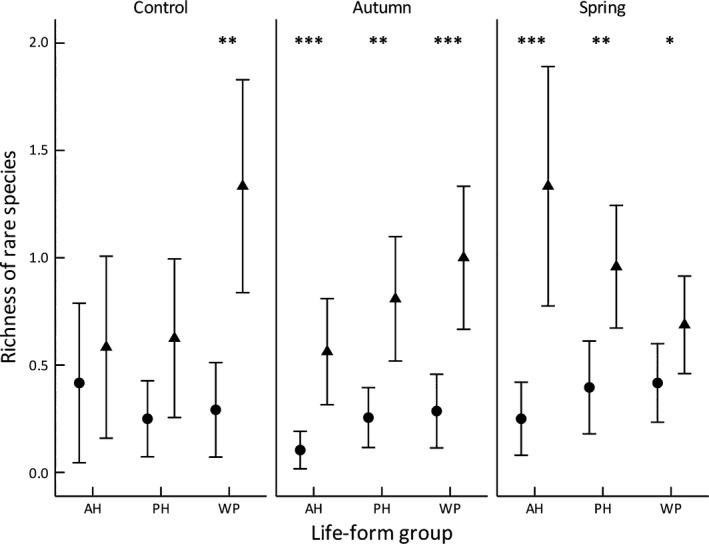
Mean (±1.96 *SE*) richness of rare species per plot in preburn (● 2010) and postburn (▲ 2013) landscapes, paneled by burn season. AH, annual herb; PH, perennial herbs or geophytes; WP, woody perennials. Wilcoxon signed ranks tests were used to compare the richness of each life‐form group between survey years (full results of tests are provided in Table S4); *p < .05, **p <* *.01, ***p <* *.001

There was a low level of similarity in the composition of rare species within landscapes grouped by burn treatment (Table 4). In unburnt control landscapes, the similarity in floristic composition of rare species was 1.8% in 2010 and 5.0% in 2013. The contribution of rare “woody perennial” species to this similarity increased from 12.4% (preburn) to 70.9% (postburn) across the survey years (Table 4), as did the richness of rare woody perennials in these unburnt landscapes (Figure 6, Table S4). For autumn‐burn landscapes, the relative contributions of each plant life‐form group to the floristic similarity of landscapes did not change markedly between 2010 and 2013, although the mean richness of rare species increased across all life‐form groups (Figure 6, Table S4). For spring‐burn landscapes, floristic similarity of rare species was greatest in 2013 (12% similarity) compared with 2010 (1.8%). Most of the similarity in rare species among spring‐burn landscapes in 2010 was explained by woody perennials (56%), whereas in 2013 it was explained by the combined contribution of perennial herb and annual herb species (40.9 and 40%, respectively; Table 4), with both of the latter increasing in species richness from 2010 to 2013 (Figure 6, Table S4).

**Table 4:** Contribution (%) of rare species in each life‐form group toward floristic similarity of rare species within (sim%), and dissimilarity (dis%) among, landscapes in preburn (2010) and postburn (2013) surveys (WP, woody perennials; PH, perennial herbs or geophytes; AH, annual herbs). Contributions of the three life‐form groups to overall (dis)similarity sum to 100%

Treatment group	Life‐form group			
	Relative contribution (%)			
	sim%	WP	PH	AH
Within 2010 surveys				
Control	1.8	12.4	43.5	44.1
Autumn	1.2	44.0	45.6	10.4
Spring	1.8	55.9	24.1	20.0
Within 2013 surveys				
Control	5.0	70.9	13.2	15.9
Autumn	6.1	53.8	33.7	12.5
Spring	12.0	20.0	40.4	39.7

Pair‐wise comparison	Life‐form group			
	Relative contribution (%)			
	dis%	WP	PH	AH
Within 2010 surveys				
Control, Autumn	99.3	34.2	36.5	29.3
Control, Spring	99.0	38.1	31.5	30.4
Autumn, Spring	98.9	44.8	36.4	18.8
Within 2013 surveys				
Control, Autumn	95.7	49.1	28.3	22.6
Control, Spring	95.6	39.3	29.5	31.2
Autumn, Spring	94.2	35.5	31.9	32.6

There was a very high level of dissimilarity in the floristic composition of rare species between burn treatments, in both survey years (Table 4). Between unburnt (control) and spring‐burn landscapes, and unburnt and autumn‐burn landscapes, the contributions to dissimilarity of rare species by each life‐form group were roughly comparable across the survey years. Autumn‐burn and spring‐burn landscapes differed more in terms of annual herb composition in 2013 than in 2010 (32.6%–18.8%, respectively).

## 4 DISCUSSION

Fire did not affect the overall floristic composition or diversity (effective number of species) of landscapes differently to the rainfall‐driven recruitment occurring in landscapes where fire was absent. Fire is known to promote the recruitment of many box–ironbark genera (Bell, 1999; Penman, Binns, Brassil, Shiels, & Allen, 2009), and so it was expected that burnt landscapes would be differentiated from unburnt landscapes by species recruiting as a consequence of fire. However, some species from these forests have seed, or a portion of seed, which will break dormancy and germinate in the absence of fire (Orscheg & Enright, 2011). Thus, it is likely that the strongest driver of species recruitment between 2010 and 2013 was the markedly above‐average rainfall acting as a cue for germination and enabling successful recruitment of nondormant seed. Drought‐conditions preceding the survey were suboptimal for rainfall‐driven germination and recruitment (Meers & Adams, 2003), and populations of species in the community were either stable or declining leading up to 2010 (Bennett et al., 2013). A soil seed bank would have developed over the years preceding the survey when environmental triggers for germination were lacking. Aside from loss of seed due to predation, it is likely that adequate propagules of many species were available (Andersen, 1989; Comino, Miller, & Enright, 2004) and awaiting environmental cues such as rainfall for germination.

Burn treatment did not affect the overall floristic composition of plots differently to sites that were unburnt. Many woody perennial and geophyte species in the community have the capacity to resprout following disturbance (Cheal, 2010; Morgan, 1999), and this would have strongly influenced similarity among landscapes and survey years. The intensity and residence time of a fire affect the ability of plants to resist fire and protect resprouting organs (Clarke et al., 2013). A fuel‐reduction burn in autumn or spring is unlikely to reach the lethal temperatures and residence time necessary to kill the dominant eucalypts (Wesolowski, Adams, & Pfautsch, 2014), particularly where fires are actively managed to be patchy. It is likely that many other resprouting species would be able to survive such fires. Species that resprout are generally poor recruiters from seed following disturbance compared to species that are obligate seeders (Paula & Pausas, 2008). With most species surviving a fire, the overall postfire species composition of dry sclerophyll forest is predominantly determined by the prior community (Christensen, Recher, & Hoare, 1981; Purdie & Slatyer, 1976).

Prescribed burns affected the type of life‐forms of rare species present in a box–ironbark forest landscape. An increase in the frequency of rare annual herbs occurring in landscapes burnt in spring compared with autumn is not surprising, as a larger proportion of the landscape was burnt in spring, potentially opening up niches and reducing competition from established plants. Propagules of annual species either found in the soil seed bank or migrating into the area (Bell et al. 1993) could take advantage of reduced competition, and increased nutrient loads in the ash bed (Certini, 2005). Such a pattern has been observed previously in other sclerophyll forests (Purdie & Slatyer, 1976), with postfire ephemerals reaching their highest cover immediately after fire, decreasing as time since fire increased (Gosper, Yates, & Prober, 2012). The lower recruitment of rare annuals in unburnt and the lesser extent autumn‐burn landscapes could be explained, at least in part, by higher competition for resources such as space, nutrients, and water, especially when common annual and ground‐cover species are filling potential niches.

Recruitment of rare woody‐perennial species was comparable between burnt and unburnt landscapes. Many species are known to require fire to trigger seed germination (Table S5), and it was anticipated that the recruitment of such species would be limited to burnt landscapes. However, species in genera that have a known postfire germination response, such as *Dillwynia* Sm. and *Pultenaea* Sm. (Auld, 1996), recruited in both burnt and unburnt landscapes (Table S2), suggesting that rainfall had the strongest effect on recruitment for many woody species. Some rare woody species with no known fire response, for example *Stenanthera pinifolia* R.Br., recruited only in unburnt sites. By conducting a prescribed burn, there is potential for negatively impacting species that are fire‐intolerant or require long‐unburnt conditions to recruit or persist (e.g., Morrison et al., 1995; Peterson & Reich, 2008).

The response of rare species in autumn‐burn landscapes could be attributed to the landscape heterogeneity produced by the patchy and relatively cool prescribed burns at that time. It is likely that a mosaic of burnt niches for annuals, unburnt refugia for fire‐intolerant species, and effects of fire to stimulate flowering and seed germination provide the greatest potential for maximizing species recruitment.

Of interest, but impossible under experimental conditions for public safety, is the effect of landscape‐scale summer fires of high intensity; further research following natural summer wildfires in these landscapes is warranted. It can be speculated that a summer fire could pose a challenge for species where resprouting ability is contingent on wildfire intensity, but potentially produce greater effects of soil heating on germination of deeply buried seed. Greater residence time allows deeper penetration of heat into the soil that could reach the seed of obligate seeder species that are deeply buried, stimulating more germination (e.g., Hindrum, Hovenden, Neyland, & Baker, 2012). This, combined with greater mortality in species that resprout, may lead to overall floristic differences between summer‐burn sites and the sites observed in this study, at least temporarily. The effect of fire frequency on rare species and floristic composition also is an important consideration for vegetation management and should be considered as part of a longer‐term research focus on the landscapes studied, as well as the effect of disturbance on grasses, orchids, and *Arthropodium*, which were not included in this study.

Recruitment of rare annual species following prescribed burns in spring follows the CSR (competitive, stress‐tolerant, and ruderal) theory of Grime (1974), insofar as the presence of ruderal annual species probably was promoted by release from competition with dominant and stress‐tolerant species. It also lends weight‐to‐mass ratio hypothesis in this system (Grime, 1998), which posits that diversity peaks soon after disturbance, as rare or less‐common species take advantage of temporary gaps within niches that are available due to a reduction in abundance of dominant species. Under this hypothesis, as highly competitive dominants recover, they modify ecosystem conditions to favor their own existence, resulting in a decline in species diversity as a proportion of species become competitively excluded with time. The functional contributions of rare and less‐common species, while important, are generally thought to be confined to facilitating the recruitment of common dominants (Grime, 1998).

Indeed, rare species were less influential than common and less‐common species in determining floristic similarity among both pre‐ and postfire landscapes and, in terms of productivity, likely play a minor role compared to larger, long‐lived dominant species. However, potentially important short‐term ecosystem roles for rare annual species exist at both the individual species and collective level. These include facilitating the recovery and recruitment of more common or long‐lived species (Connell & Slatyer, 1977; Soliveres, Smit, & Maestre, 2014) or playing roles in soil stabilization (Pohl, Alig, Körner, & Rixen, 2009), nutrient dynamics (Marsh et al., 2000), water dynamics (Rixen & Mulder, 2005), providing supplementary or novel resources for biota (Swanson et al., 2010), or having below‐ground effects on soil biota (Mariotte, 2014).

Prescribed burning in fire‐tolerant ecosystems can promote the recruitment of species that are rare within a landscape, but the context of a disturbance (e.g., season, extent, and postfire weather) can dictate their response. Managing disturbance to produce heterogeneous environments may be most beneficial for maintaining a diversity of rare species' life‐forms on a landscape scale. This in turn can promote functional diversity, which can provide benefits in buffering and stabilizing ecosystem processes in the face of future disturbance.

## CONFLICT OF INTEREST

None declared.

## AUTHORS' CONTRIBUTIONS

AFB, MFC, and GJH conceived ideas and designed methodology for data collection; JP, MD, and MG conceived ideas and designed methodology for data analysis; GJH coordinated all field activities; EB collected the data; GJH and KC were responsible for data curation; JP and MD analyzed the data; JP, MD, MG, and TW led the writing of the manuscript. All authors contributed critically to the draft and gave final approval for publication.

## DATA ACCESSIBILITY

Data associated with this paper are available online at: https://doi.org/10.6084/m9.figshare.5143156.v1.
