# Supplementary material for: The effect of prescribed burning on plant rarity in a temperate forest
Source: Ecol Evol. 2018 Jan 8;8(3):1714–25. doi: 10.1002/ece3.3771 (PMC5792504; doi:10.1002/ece3.3771)
Supplement: Supplementary file 1 [file ECE3-8-1714-s001.docx]

**Table S1.** Permutational analysis of dispersions among plots based on their floristic composition, with year of survey and season of burn as fixed factors. Comparisons including all species in the community, as well as species life-form and frequency groups, are shown. Significant results are provided in bold.

| **Group** | ***Year*** | | ***Burn*** | | ***Year*burn*** | | |  |
| --- | --- | --- | --- | --- | --- | --- | --- | --- |
|  | ***F*_(1, 238)_** | ***P*** | ***F*_(2,237)_** | ***P*** | | ***F*_(5, 234)_** | ***P*** | |
| All species | 0.23 | 0.650 | 2.56 | 0.096 | | 1.66 | 0.197 | |
| *Life-form* |  |  |  |  | |  |  | |
| Woody perennials | <0.01 | 0.990 | 1.62 | 0.240 | | 1.03 | 0.475 | |
| Perennial herbs or geophytes | 2.91 | 0.112 | 1.97 | 0.175 | | 2.03 | 0.122 | |
| Annual herbs | 10.52 | **0.005** | 1.04 | 0.465 | | 3.61 | **0.020** | |
| *Frequency* |  |  |  |  | |  |  | |
| Common | 1.36 | 0.266 | 0.85 | 0.470 | | 0.80 | 0.610 | |
| Less-common | 13.74 | **<0.001** | 5.01 | **0.010** | | 6.57 | **<0.001** | |
| Rare | 95.41 | **<0.001** | 2.21 | 0.266 | | 19.81 | **<0.001** | |

**Table S2.** Pairwise comparisons of average dispersion distance (distance-to-centroid, calculated by permutational analysis of dispersions) among plots within different treatment groups (survey year, season of prescribed burn). Significant results are provided in bold.

| **Pairwise comparisons** | ***Annual herbs*** | | ***Less common*** | | ***Rare*** | |
| --- | --- | --- | --- | --- | --- | --- |
|  | ***t*** | ***P*** | ***t*** | ***P*** | ***t*** | ***P*** |
| *Within 2010 surveys* |  |  |  |  |  |  |
| Control, Autumn | 2.14 | 0.062 | 3.37 | **0.003** | 1.27 | 0.309 |
| Control, Spring | 0.81 | 0.503 | 2.79 | **0.010** | 0.39 | 0.767 |
| Autumn, Spring | 1.73 | 0.139 | 0.46 | 0.666 | 1.95 | 0.144 |
| *Within 2013 surveys* |  |  |  |  |  |  |
| Control, Autumn | 0.51 | 0.679 | 1.64 | 0.128 | 0.42 | 0.733 |
| Control, Spring | 0.78 | 0.529 | 1.50 | 0.169 | 0.07 | 0.954 |
| Autumn, Spring | 0.34 | 0.785 | 0.09 | 0.933 | 0.48 | 0.675 |
| *Between 2010 and 2013* |  |  |  |  |  |  |
| Control | 0.01 | 0.987 | 3.99 | **<0.001** | 4.38 | **<0.001** |
| Autumn | 3.29 | **0.003** | 1.86 | 0.077 | 6.97 | **<0.001** |
| Spring | 1.95 | 0.099 | 2.18 | **0.038** | 5.15 | **<0.001** |
| *Within burn treatments* |  |  |  |  |  |  |
| Control, Autumn | 0.48 | 0.677 | 3.12 | **0.003** | 0.94 | 0.480 |
| Control, Spring | 0.69 | 0.559 | 2.66 | **0.011** | 0.78 | 0.541 |
| Autumn, Spring | 1.42 | 0.222 | 0.51 | 0.632 | 2.08 | 0.108 |

**Table S3.** Average (± SE) dispersion distance (distance-to-centroid, calculated by permutational analysis of dispersions) among plots within different treatment groups (survey year, season of prescribed burn). Burn treatments are pooled when comparing effect of survey year, and survey years are pooled when comparing effect of burn treatments.

| **Treatment group** | **Herbs** | |  | **Less-common** | |  | **Rare** | |
| --- | --- | --- | --- | --- | --- | --- | --- | --- |
|  | **Average** | **SE** |  | **Average** | **SE** |  | **Average** | **SE** |
| 2010 | 34.92 | 0.83 |  | 44.47 | 0.83 |  | 24.99 | 1.72 |
| 2013 | 32.93 | 0.82 |  | 40.53 | 0.67 |  | 44.60 | 1.04 |
|  |  |  |  |  |  |  |  |  |
| Control 2010 | 35.19 | 2.11 |  | 49.07 | 1.45 |  | 26.56 | 3.35 |
| Control 2013 | 35.24 | 2.12 |  | 41.72 | 1.14 |  | 43.92 | 2.11 |
| Autumn 2010 | 29.04 | 1.73 |  | 42.09 | 1.27 |  | 21.10 | 2.55 |
| Autumn 2013 | 36.58 | 1.50 |  | 39.10 | 0.98 |  | 42.69 | 1.76 |
| Spring 2010 | 33.05 | 1.55 |  | 42.95 | 1.37 |  | 28.23 | 2.68 |
| Spring 2013 | 37.31 | 1.54 |  | 39.23 | 1.03 |  | 43.76 | 1.33 |
|  |  |  |  |  |  |  |  |  |
| Control | 35.82 | 1.38 |  | 46.15 | 1.12 |  | 37.03 | 2.19 |
| Autumn | 35.02 | 0.97 |  | 41.61 | 0.86 |  | 34.29 | 1.75 |
| Spring | 37.08 | 1.07 |  | 42.23 | 0.88 |  | 39.01 | 1.50 |

**Table S4.** Difference in richness of rare species from different plant life-form groups within autumn-burn, spring-burn and unburnt control plots, before (2010) and after (2013) prescribed burning (AH, annual herb; PH, perennial herb or geophyte; WP, woody perennial). Significant results from Wilcoxon signed ranks tests are provided in bold.

| **Treatment group** | **AH** | |  | **PH** | |  | **WP** | |
| --- | --- | --- | --- | --- | --- | --- | --- | --- |
|  | **Z** | ***P*** |  | **Z** | ***P*** |  | **Z** | ***P*** |
| Control | -0.65 | 0.518 |  | -1.81 | 0.070 |  | -3.47 | **0.001** |
| Autumn | -4.27 | **<0.001** |  | -3.29 | **0.001** |  | -4.09 | **<0.001** |
| Spring | -4.44 | **<0.001** |  | -3.45 | **0.001** |  | -2.20 | **0.028** |

**Table S5.** List of rare species and the number of plots in which they were detected within a box-ironbark forest before (10 = 2010) and after (13 = 2013) prescribed burning in different seasons (U = unburnt, A = autumn, S = spring). Broad fire response groups of species are included in column Re (can resprout after fire) and Se (heat or smoke stimulated germination); references are provided as a footnote. ? = fire response undocumented.

| **Woody perennials** |  |  |  |  |  |  |  |  |  |
| --- | --- | --- | --- | --- | --- | --- | --- | --- | --- |
|  | **U** | | **A** | | **S** | | **Re** | **Se** |  |
| **Species** | **10** | **13** | **10** | **13** | **10** | **13** |  |  |  |
|  |  |  |  |  |  |  |  |  |  |
| *Eucalyptus goniocalyx* F.Muell. ex Miq. | 2 | 2 | - | - | 1 | 1 | ? | ✓^1^ |  |
| *Hovea heterophylla* A.Cunn. ex Hook.f. | 1 | 5 | 1 | 6 | 1 | 2 | ✓^2^ | ✓^1^ |  |
| *Exocarpos cupressiformis* Labill. | 1 | 3 | 1 | 2 | 1 | - | ✓^3^ | x^1^ |  |
| *Amyema miquelii* (Lehm. ex Miq.) Tiegh. | 1 | 2 | - | 1 | - | 1 | ✓^4^ | x^1^ |  |
| *Melichrus urceolatus* R.Br. | 1 | 2 | - | - | - | - | ✓^3^ | ? |  |
| *Calytrix tetragona* Labill. | 1 | 1 | 2 | 1 | - | - | ✓^2^ | ? |  |
| *Dillwynia sericea* A.Cunn. | - | 5 | 2 | 12 | 1 | 8 | ? | ✓^1^ |  |
| *Pultenaea pedunculata* Hook. | - | 4 | - | 3 | - | 1 | ? | ✓^1^ |  |
| *Pimelea humilis* R.Br. | - | 3 | - | - | 5 | 10 | ✓^5^ | ? |  |
| *Dillwynia cinerascens* R.Br. ex Sims | - | 2 | 1 | 1 | - | 1 | ? | ✓^1^ |  |
| *Eucalyptus melliodora* A.Cunn. ex Schauer | - | 1 | - | - | 1 | - | ✓^6^ | ✓^1^ |  |
| *Pultenaea graveolens* Tate | - | 1 | - | - | - | 1 | ? | ✓^1^ |  |
| *Stenanthera pinifolia* R.Br. | - | 1 | - | - | - | - | X^7^ | x^1^ |  |
| *Acacia gunnii* Benth. | - | - | 4 | 5 | 1 | 2 | ✓^8^ | ✓^1^ |  |
| *Boronia anemonifolia* A.Cunn. | - | - | 1 | 8 | - | - | ✓^9^ | ? |  |
| *Prostanthera denticulata* R.Br. | - | - | 1 | 3 | 1 | 1 | ? | ✓^1^ |  |
| *Hardenbergia violacea* (Schneev.) Stearn | - | - | 1 | 2 | 1 | 3 | ✓^3^ | ✓^1^ |  |
| *Acacia montana* Benth. | - | - | - | 2 | 4 | 1 | ? | ✓^1^ |  |
| *Gompholobium huegelii* Benth. | - | - | - | 2 | - | - | ✓^7^ | ✓^1^ |  |
| *Hakea decurrens* R.Br. | - | - | - | 1 | - | - | x^10^ | ✓^1^ |  |
| *Philotheca verrucosa* (A.Rich.) Paul G. Wilson | - | - | - | - | 2 | 1 | ? | ? |  |
| *Indigofera australis* Willd. subsp. a*ustralis* | - | - | - | - | 1 | - | ✓^3,8^ | ✓^1^ |  |

**Table S5 (contd.).**

| **Perennial herbs or geophytes** |  |  |  |  |  |  |  |  | |  |
| --- | --- | --- | --- | --- | --- | --- | --- | --- | --- | --- |
|  | **U** | | **A** | | **S** | | **Re** | | **Se** | |
| **Species** | **10** | **13** | **10** | **13** | **10** | **13** |  |  | |  |
|  |  |  |  |  |  |  |  |  | |  |
| *Stylidium graminifolium* sensu Willis (1972) | 2 | 2 | 1 | 8 | 1 | 3 | ✓^3,8^ | ✓^1^ | |  |
| *Juncus subsecundus* N.A.Wakef. | 2 | - | 1 | - | 2 | 1 | ? | x^1^ | |  |
| *Leptorhynchos tenuifolius* F.Muell. | 1 | 1 | - | - | - | - | ? | ? | |  |
| *Gonocarpus elatus* (A.Cunn. ex Fenzl) Orchard | 1 | - | - | - | - | - | ✓^11^ | ✓^1^ | |  |
| *Lobelia gibbosa* Labill. | - | 4 | 2 | 1 | 4 | - | ✓^12^ | ? | |  |
| *Lagenophora huegelii* Benth. | - | 2 | 2 | - | 3 | - | ? | x^1^ | |  |
| *Cheilanthes austrotenuifolia* H.M.Quirk & T.C. Chambers | - | 2 | 1 | 1 | 1 | 1 | ✓^11,13^ | x^1^ | |  |
| *Drosera glanduligera* Lehm. | - | 1 | 2 | 5 | - | - | ? | ? | |  |
| *Galium gaudichaudii* DC. | - | 1 | - | 5 | 2 | 1 | ? | x^1^ | |  |
| *Senecio hispidulus* A.Rich. | - | 1 | - | - | 1 | 3 | x^8,14^ | x^1^ | |  |
| *Stypandra glauca* R.Br. | - | 1 | - | - | 1 | 1 | ✓^12^ | ? | |  |
| *Brachyscome multifida* DC. | - | - | 3 | 2 | 2 | 3 | ? | x^1^ | |  |
| *Senecio quadridentatus* Labill. | - | - | - | 6 | 1 | 20 | x^8,13^ | x^1^ | |  |
| *Brachyscome perpusilla* (Steetz) J.M.Black | - | - | - | 3 | - | - | ? | x^1^ | |  |
| *Burchardia umbellata* R.Br. | - | - | - | 3 | - | - | ✓^15^ | ? | |  |
| *Drosera peltata* sensu Conn (1996) | - | - | - | 2 | - | - | ✓^5,7^ | x^5^ | |  |
| *Epilobium billardiereanum* Ser. ex DC. | - | - | - | 1 | - | 2 | ✓^12^ | x^1^ | |  |
| *Plantago hispida* R.Br. | - | - | - | 1 | - | - | ? | x^1^ | |  |
| *Leptorhynchos squamatus* (Labill.) Less. | - | - | - | - | 1 | - | ✓^5,8^ | x^1^ | |  |
| *Euchiton japonicus* (Thunb.) Holub | - | - | - | - | - | 3 | ? | x^1^ | |  |
| *Euchiton sphaericus* (Willd.) Holub | - | - | - | - | - | 3 | x^7,13^ | x^1^ | |  |
| *Senecio runcinifolius* J.H.Willis | - | - | - | - | - | 3 | ? | x^1^ | |  |
| *Lepidosperma laterale* R.Br. | - | - | - | - | - | 1 | ✓^16^ | ? | |  |
| *Luzula meridionalis* Nordensk. | - | - | - | - | - | 1 | ✓^5,8^ | ? | |  |
|  |  |  |  |  |  |  |  |  | |  |

**Table S5 (contd.).**

| **Annual herbs** |  |  |  |  |  |  |  |  |
| --- | --- | --- | --- | --- | --- | --- | --- | --- |
|  | **U** |  | **A** |  | **S** |  | **Re** | **Se** |
| **Species** | **10** | **13** | **10** | **13** | **10** | **13** |  |  |
|  |  |  |  |  |  |  |  |  |
| *Calandrinia calyptrata* Hook.f. | 3 | 3 | 2 | 4 | - | 2 | ? | ? |
| *Crassula sieberiana* sensu Toelken, Jeanes & Stajsic (1996) | 2 | 3 | - | 4 | 2 | 16 | x^3,17^ | ✓^1^ |
| *Poranthera microphylla* Brongn. | 2 | 3 | - | - | 3 | 4 | x^12^ | ✓^1^ |
| *Siloxerus multiflorus* Nees | 2 | 1 | - | 4 | 1 | 1 | ? | ? |
| *Stuartina muelleri* Sond. | 1 | - | - | 1 | 4 | 4 | ? | x^1^ |
| *Centrolepis strigosa* (R.Br.) Roem. & Schult. subsp. *strigosa* | - | 2 | 1 | 1 | 1 | 3 | ✓^12^ | ✓^12^ |
| *Crassula decumbens* Thunb. | - | 1 | - | 4 | 1 | 13 | x^18^ | ✓^1^ |
| *Hyalosperma demissum* (A.Gray) Paul G.Wilson | - | 1 | - | 2 | - | 2 | ? | ? |
| *Juncus bufonius* L. | - | - | 2 | - | - | 2 | ? | ? |
| *Hydrocotyle foveolata* H.Eichler | - | - | - | 5 | - | 8 | ? | ? |
| *Crassula colorata* (Nees) Ostenf. | - | - | - | 1 | - | 4 | ? | ✓^1^ |
| *Crassula peduncularis* (Sm.) Meigen | - | - | - | 1 | - | - | ? | ✓^1^ |
| *Senecio biserratus* Belcher | - | - | - | - | - | 2 | ? | ? |
| *Gnaphalium indutum* Hook.f. | - | - | - | - | - | 1 | ? | ? |
| *Senecio glomeratus* Desf. ex Poir. | - | - | - | - | - | 1 | ? | ? |
| *Triptilodiscus pygmaeus* Turcz. | - | - | - | - | - | 1 | ? | ✓^1^ |
|  |  |  |  |  |  |  |  |  |

^1^ Ralph, M. (2003) and references therein; *Growing Australian native plants from seed*.

Murray Ralph / Bushland Horticulture, Fitzroy.

^2^ Vivian, L.M. & Cary, G.J. (2011) Relationship between leaf traits and fire-response strategies in

shrub species of a mountainous region of south-eastern Australia. *Annals of Botany*, **109(1),** 197-208.

^3^ Purdie, R.W. & Slatyer, R.O. (1976) Vegetation Succession after Fire in Sclerophyll

Woodland Communities in South‐Eastern Australia. *Australian Journal of Ecology*, **1(4),** 223-236.

^4^ Kelly, P., Reid, N. & Davis, I. (1997) Effects of Experimental Burning, Defoliation, and

Pruning on Survival and Vegetative Resprouting in Mistletoes (*Amyema miquelii* and *Amyema pendula*). *International Journal of Plant Sciences*, **158(6),** 856-861.

^5^ Morgan, J.W. (1999) Defining grassland fire events and the response of perennial plants to

annual fire in temperate grasslands of south-eastern Australia. *Plant Ecology*, **144,** 127-144.

^6^ Denham, A.J., Vincent, B.E., Clarke, P.J. & Auld, T.D. (2016) Responses of tree species to a

severe fire indicate major structural change to Eucalyptus–Callitris forests. *Plant Ecology*, **217,** 617–629.

^7^ Wills, T.J. & Read, J. (2007) Soil seed bank dynamics in post-fire heathland succession in

south-eastern Australia. *Plant Ecology*, **190,** 1–12.

^8^ Kitchin, M., Wright, G., Robertson, G., Brown, D., Tolsma, A. & Stern, S.E. (2013) *Long term*

*monitoring for Fire Management – 10 years on for the Australian Alps fire plots; Technical Report No 26.* Environment and Sustainable Development Directorate, ACT Government.

^9^ Benson, D. & McDougall, L. (2001) Ecology of Sydney plant species part 8: dicotyledon

families Rutaceae to Zygophyllaceae. *Cunninghamia*, **7(2),** 241-462.

^10^ Enright, N.J. & Goldblum, D. (1999) Demography of a non-sprouting and resprouting Hakea

species (Proteaceae) in fire-prone Eucalyptus woodlands of southeastern Australia in relation to stand age, drought and disease. *Plant Ecology*, **144(1),** 71-82.

^11^ Vesk, P.A., Warton, D.I. & Westoby, M. (2004) Sprouting by semi-arid plants: testing a

dichotomy and predictive traits. *Oikos*, **107,** 72-89.

^12^ Penman, T.D., Binns, D., Allen, R., Shiels, R. & Plummer, S. (2008) Germination responses of a

dry sclerophyll forest soil-stored seedbank to fire related cues. *Cunninghamia*, **10,** 547-555.

^13^ Downe, J. & Coates, F. (2004) *Recovery of Silurian Limestone Pomaderris Shrubland after*

*the 2003 bushfires in north-east Victoria; Arthur Rylah Institute for Environmental Research Technical Report No. 151.* Department of Sustainability and Environment, Victoria.

^14^ Prober, S.M., Thiele, K.R. & Bramwell, M. (2007) Intense fires promote uncommon fire

ephemerals in Currawang *Acacia doratoxylon* dry scrubs of Little River Gorge, East Gippsland. *Victorian Naturalist*, **124(6),** 320-331.

^15^ Allen, T., Brewster, E., Brown, M., Drummond, D., Elli, M., Gurling, J., Laby, R.J., Wallis, G.,

Burrows, F. & Jones, J. (2008) Notes on the post-fire recovery of plants at Wilsons Promontory. *Victorian Naturalist*, **125(3)*,*** 87-91.

^16^ Benson, D. & McDougall, L. (2002) Ecology of Sydney plant species part 9:

Monocotyledon families Agavaceae to Juncaginaceae. *Cunninghamia*, **7(4),** 695-930.

^17^ Venn, S.E. & Morgan, J.W. (2010) Soil seedbank composition and dynamics across alpine

summits in south-eastern Australia. *Australian Journal of Botany*, **58,** 349-362.

^18^ Benson, D. & McDougall, L. (1995) Ecology of Sydney plant species part 3: dicotyledon

families Cabombaceae to Eupomatiaceae. *Cunninghamia*, **4(2),** 789-100


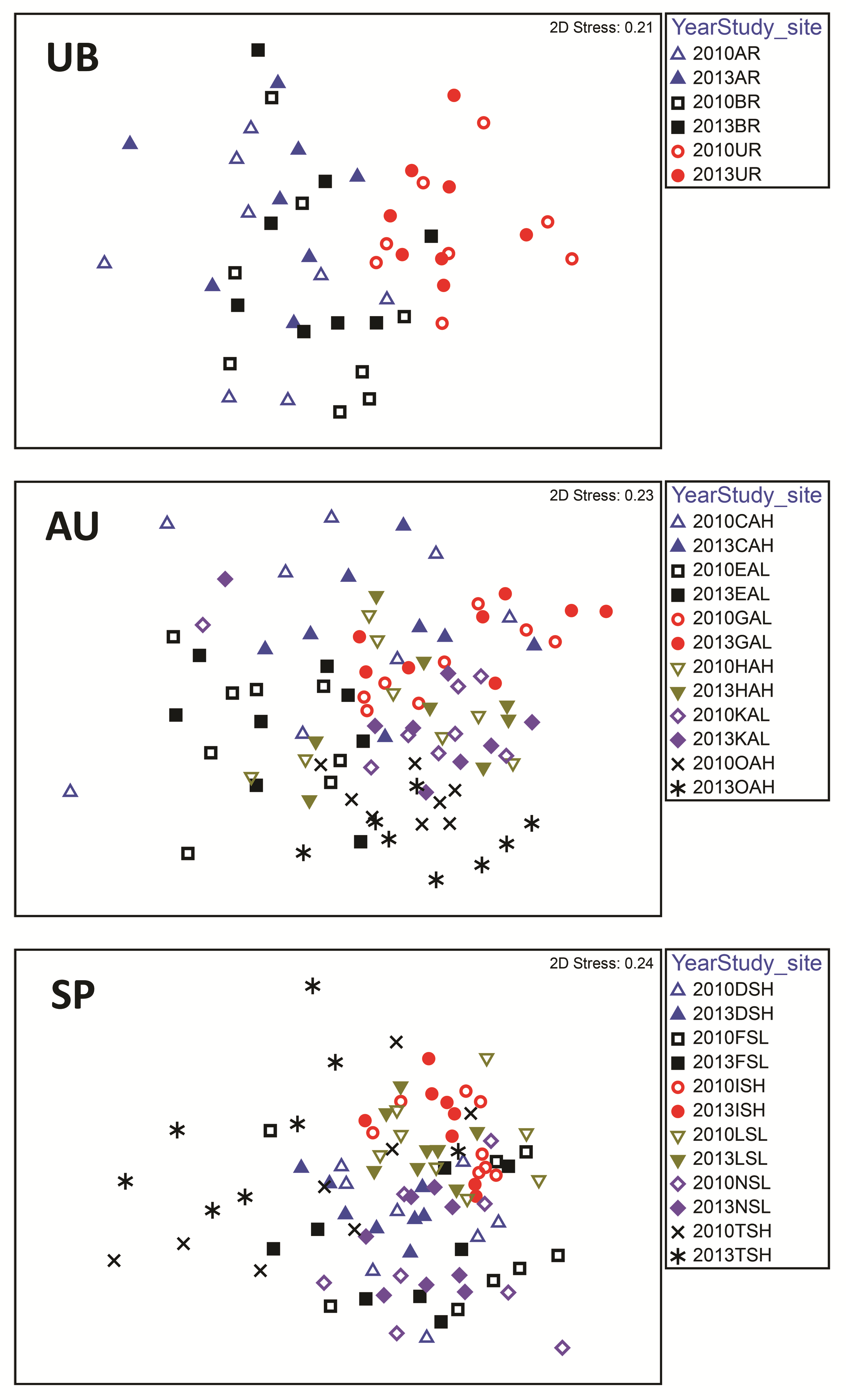


**Fig. S1.** Similarity of floristic composition (nMDS, Bray Curtis similarity) in sites within landscapes before (2010, open symbols), and after (2013, closed symbols) prescribed burn treatments in autumn (AU), spring (SP) or left unburnt as a control (UB). ‘YearStudy_site’ displays the code for each landscape, and their corresponding symbol in the plot.


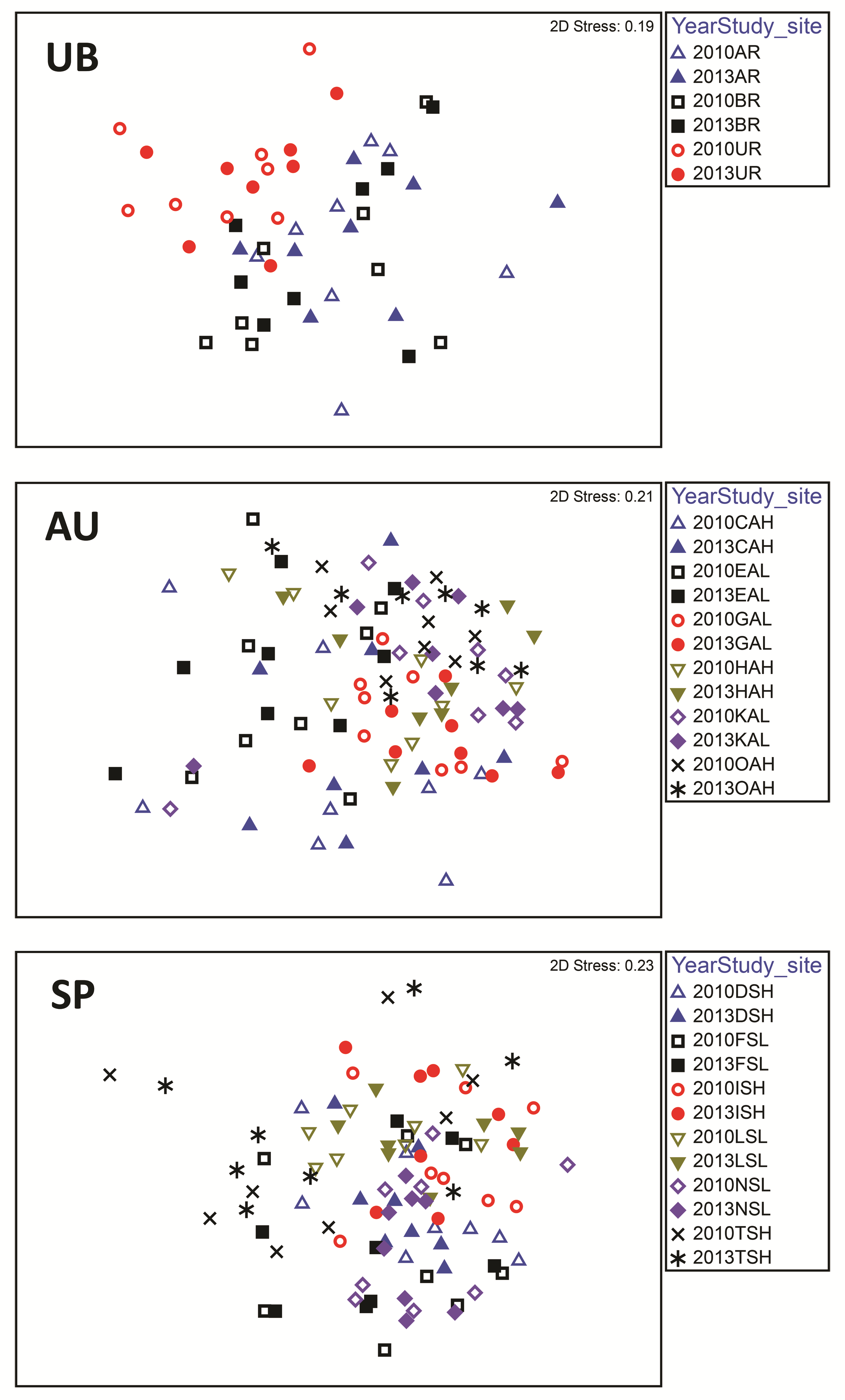


**Fig. S2.** Similarity of floristic composition (nMDS, Bray Curtis similarity) of common species in sites within landscapes before (2010, open symbols), and after (2013, closed symbols) prescribed burn treatments in autumn (AU), spring (SP) or left unburnt as a control (UB). ‘YearStudy_site’ displays the code for each landscape, and their corresponding symbol in the plot.


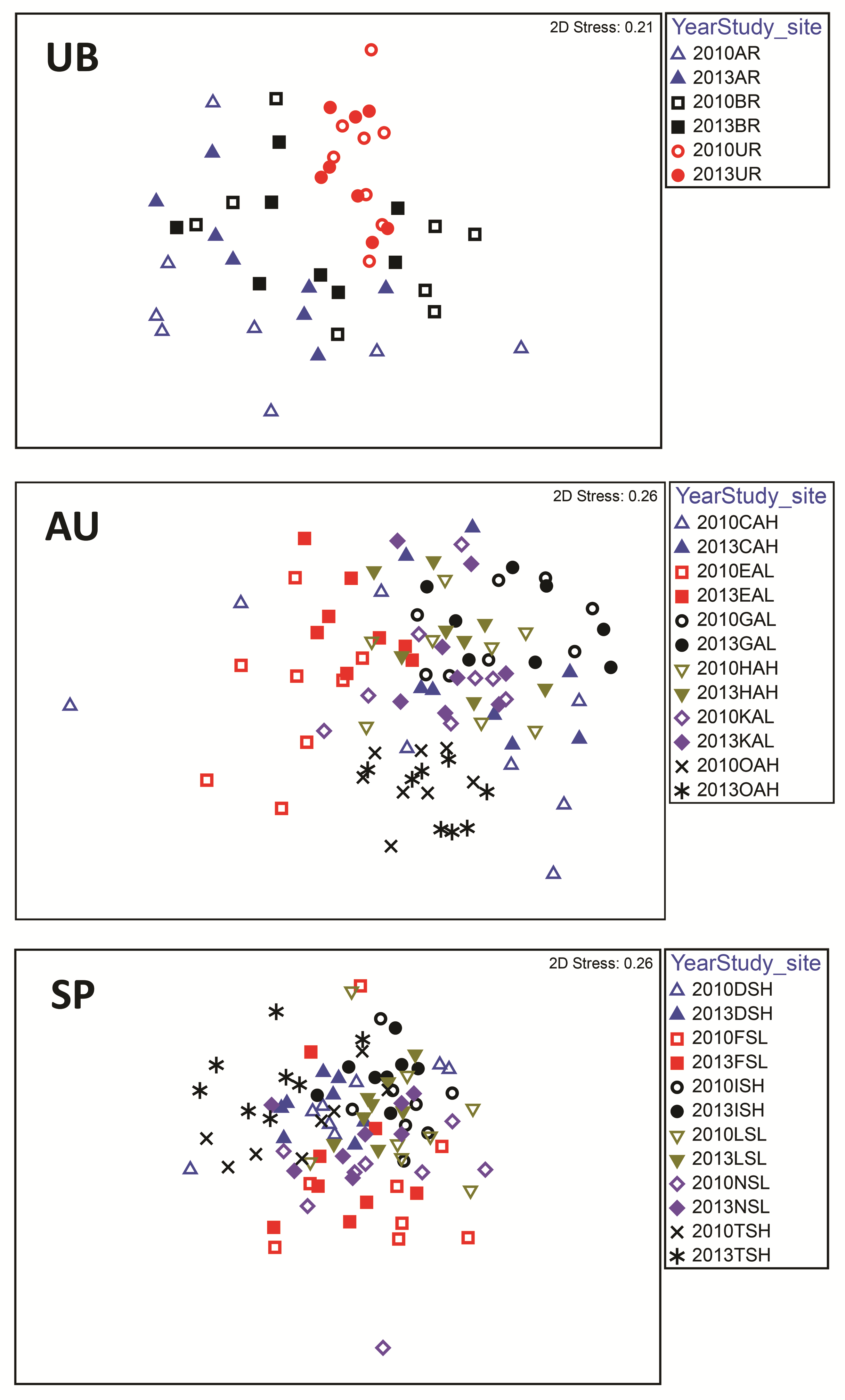


**Fig. S3.** Similarity of floristic composition (nMDS, Bray Curtis similarity) of less-common species in sites within landscapes before (2010, open symbols), and after (2013, closed symbols) prescribed burn treatments in autumn (AU), spring (SP) or left unburnt as a control (UB). ‘YearStudy_site’ displays the code for each landscape, and their corresponding symbol in the plot.


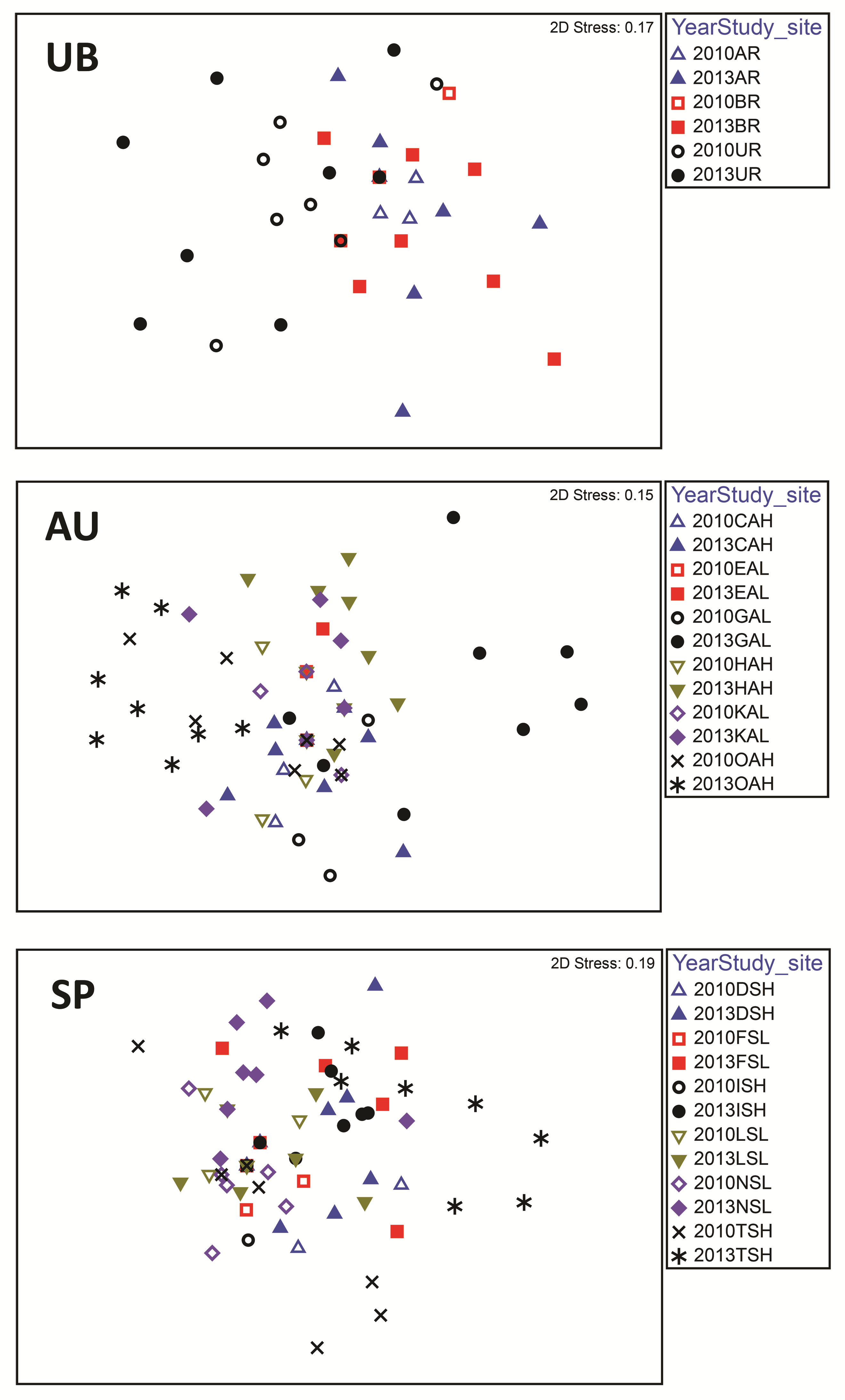


**Fig. S4.** Similarity of floristic composition (nMDS, Bray Curtis similarity) of rare species in sites within landscapes before (2010, open symbols), and after (2013, closed symbols) prescribed burn treatments in autumn (AU), spring (SP) or left unburnt as a control (UB). ‘YearStudy_site’ displays the code for each landscape, and their corresponding symbol in the plot.


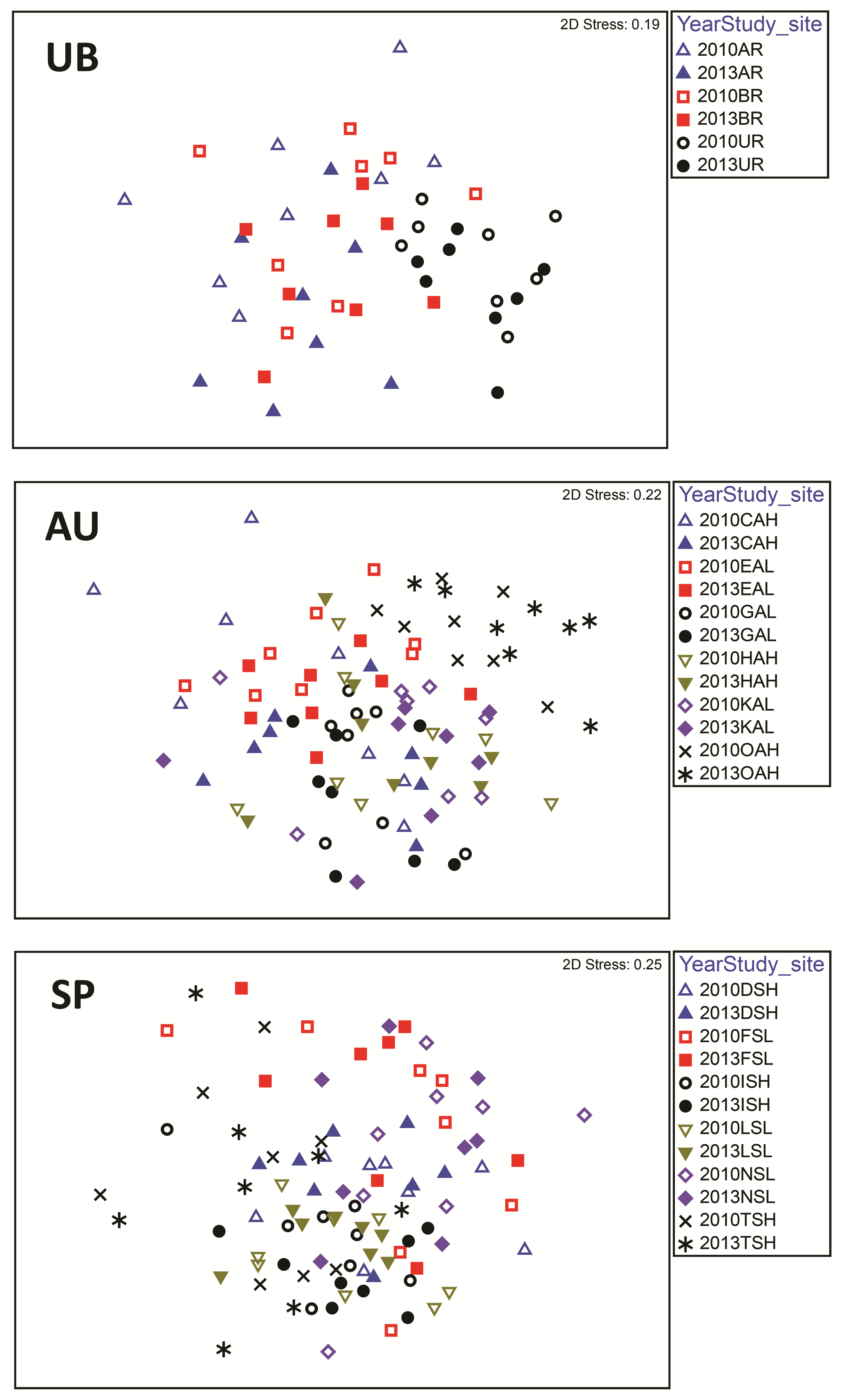


**Fig. S5.** Similarity of floristic composition (nMDS, Bray Curtis similarity) of woody perennial species in sites within landscapes before (2010, open symbols), and after (2013, closed symbols) prescribed burn treatments in autumn (AU), spring (SP) or left unburnt as a control (UB). ‘YearStudy_site’ displays the code for each landscape, and their corresponding symbol in the plot.


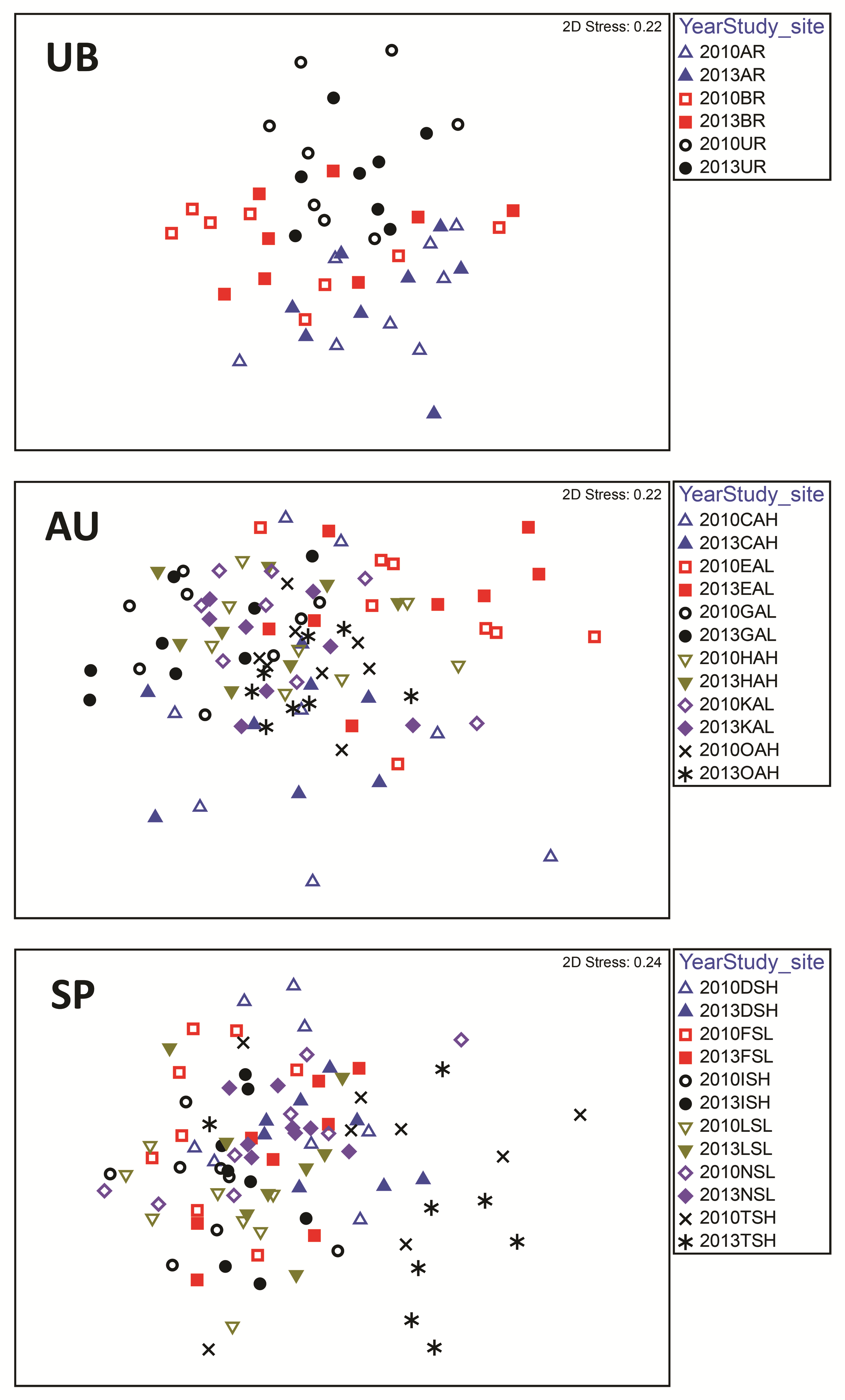


**Fig. S6.** Similarity of floristic composition (nMDS, Bray Curtis similarity) of perennial herb and geophyte species in sites within landscapes before (2010, open symbols), and after (2013, closed symbols) prescribed burn treatments in autumn (AU), spring (SP) or left unburnt as a control (UB). ‘YearStudy_site’ displays the code for each landscape, and their corresponding symbol in the plot.


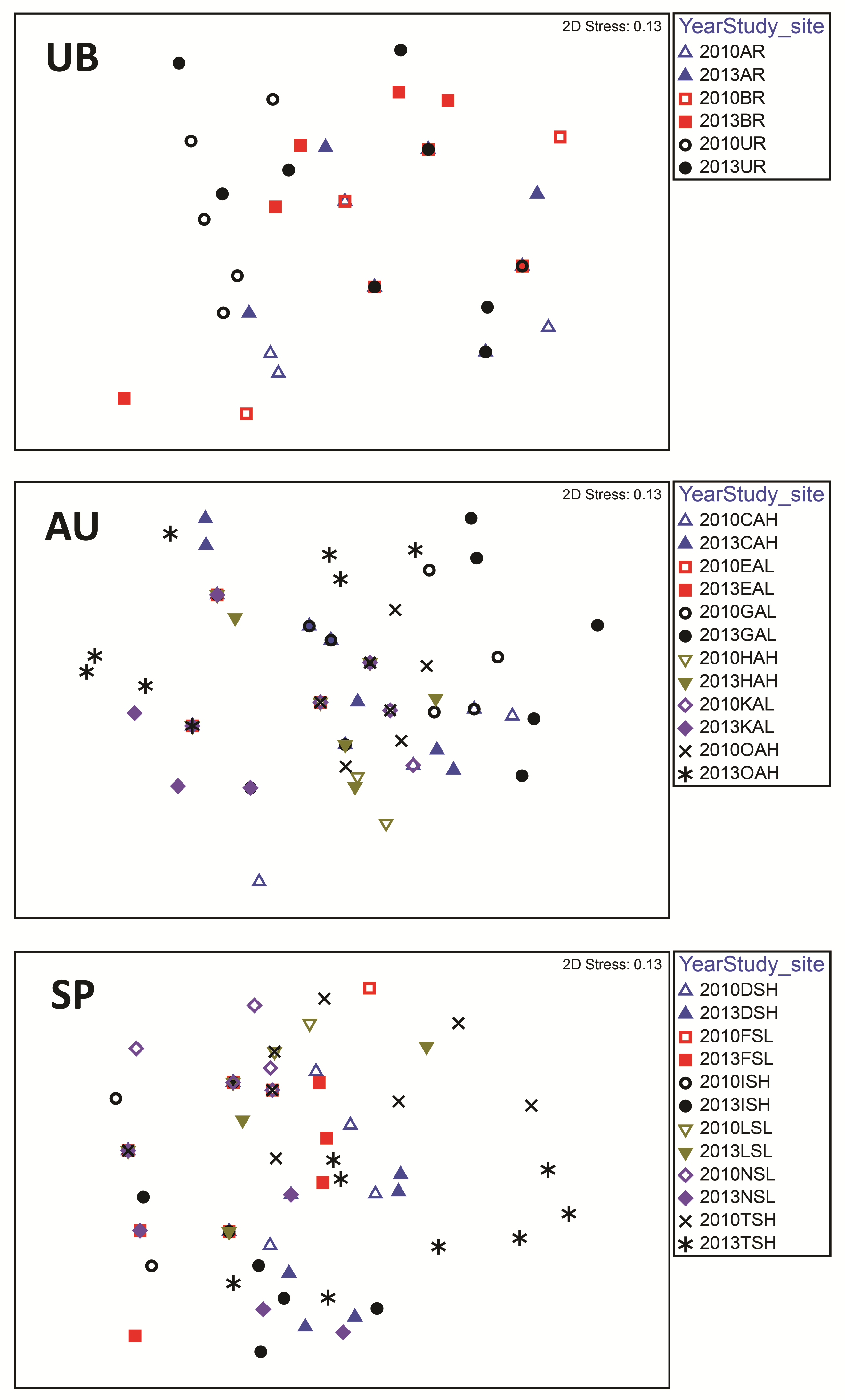


**Fig. S7.** Similarity of floristic composition (nMDS, Bray Curtis similarity) of annual herb species in sites within landscapes before (2010, open symbols), and after (2013, closed symbols) prescribed burn treatments in autumn (AU), spring (SP) or left unburnt as a control (UB). ‘YearStudy_site’ displays the code for each landscape, and their corresponding symbol in the plot.
